# The Impact of Pulmonary Hypertension on Hospitalization Risk in Adults with Respiratory Syncytial Virus Infection

**DOI:** 10.3390/biomedicines13092272

**Published:** 2025-09-16

**Authors:** Mayuri Mudgal, Aseem Rai Bhatnagar, Aneesh Kumar Vasudevan, Ajeetha Priya Gajendiran, Venkatesh Gondhi, Swetha Balaji, Shanjai Krishnan Murugan, Kulothungan Gunasekaran

**Affiliations:** 1 Internal Medicine, Camden Clark Medical Center, Parkersburg, WV 26101, USA; 2 Department of Radiation Oncology, Henry Ford Health System, Detroit, MI 48202, USA; abhatna1@hfhs.org; 3 Department of Critical Care Medicine, Onvida Health, Yuma, AZ 85364, USA; avasudevan1@onvidahealth.org; 4 Pulmonary and Critical Care, Onvida Health, Yuma, AZ 85364, USA; ajeetha19@gmail.com (A.P.G.); kgunasekaran@yumaregional.org (K.G.); 5 Hospital Medicine, Onvida Health, Yuma, AZ 85364, USA; gondhivenkatesh@gmail.com; 6 Internal Medicine, St. Mary’s General Hospital, New York Medical College and St. Clare’s Health, Passaic, NJ 07055, USA; n.b.swetha@gmail.com; 7 Department of Medicine, Stanley Medical College, Chennai 600001, TN, India; shanjaikrishnan@gmail.com

**Keywords:** pulmonary hypertension, respiratory syncytial virus, hospitalization risk, RSV vaccination, pulmonary hypertension WHO groups

## Abstract

**Background/Objectives**: Respiratory syncytial virus (RSV) infection can lead to significant complications, particularly among those with underlying cardiovascular and pulmonary complications. Patients with pulmonary hypertension (PH) are susceptible to clinical deterioration triggered by respiratory infections due to their limited cardiopulmonary reserve. This study aimed to assess the risk of hospitalization in RSV-infected adults with and without PH. **Methods**: We conducted a retrospective cohort study using the research network TriNetX to assess the risk of hospitalization in a cohort of patients with RSV infection, comparing those with and without PH. Propensity score matching was performed for demographic variables and RSV risk factors between the two cohorts. The risk of hospitalization was expressed as an adjusted odds ratio (aOR) with a 95% confidence interval (CI). **Results**: There were 193,256 patients in the RSV with PH cohort and 2,843,714 in the RSV without PH cohort (all aged >18 years). The mean age of the RSV with PH cohort was 68.2 ± 15.3 years, 50.6% were females, 64% were white, and 64.2% were group 2 PH. The RSV with PH cohort was at an increased risk of hospitalization (aOR 1.89, 95% CI 1.87–1.92, *p*-value 0.02). There was a significant risk (aOR 1.29, 95% CI 1.27–1.32) for the composite outcome of hospitalization-related complications between the two cohorts. Comorbid conditions (diabetes, cardiovascular disease, chronic lung disease, and chronic kidney disease) increased the risk of hospitalization in the RSV with PH group, with the biggest effect noted with underlying cardiovascular disease. Similarly, those with group 2 PH had a higher risk of hospitalization compared to the other PH groups. Remarkably, all PH groups demonstrated increased hospitalization risk compared to the RSV without PH cohort. **Conclusions**: We found that patients >18 years of age with PH and RSV infection were at an increased risk of hospitalization, with subsequently higher rates of RSV-infection-related complications. All PH groups had a higher hospitalization risk compared to the RSV without PH cohort, likely denoting PH as an independent risk factor for worse RSV-infection-related outcomes. RSV vaccination, therefore, may benefit all age groups of patients with PH.

## 1. Introduction

Pulmonary hypertension (PH) is a chronic progressive condition characterized by increased pulmonary vascular resistance and remodeling of the pulmonary arteries, ultimately leading to right ventricular failure and premature mortality [1]. Based on the cause and mechanism, the World Health Organization-sponsored 2008 Dana Point clinical classification schema (WHO) has divided pulmonary hypertension (PH) into five categories: group 1, pulmonary arterial hypertension (PAH), comprises idiopathic, heritable, drug-induced, and connective tissue disease-associated forms; group 2 results from left heart disease, including valve problems or heart failure; group 3 consists of lung disorders or hypoxia, such as chronic obstructive pulmonary disease (COPD) or interstitial lung disease (ILD); group 4 includes unresolved clots causing chronic thromboembolic pulmonary hypertension; and, lastly, group 5 PH includes either uncertain or complex etiology [2].

Respiratory syncytial virus (RSV), a negative-sense RNA virus, is a well-established cause of severe lower respiratory tract infections in infants and young children. However, growing evidence underscores its significant disease burden in adults, particularly in older individuals and those with underlying cardiopulmonary comorbidities [3,4]. RSV infection has been associated with high morbidity, including pneumonia, acute exacerbations of chronic lung disease, and cardiovascular complications [5]. It is estimated that RSV leads to $1.2 billion in costs annually in the United States [6]. A systematic review and meta-analysis of the RSV burden in adults aged 60 years and older in high-income countries estimated that RSV-associated acute respiratory infections (ARIs) led to approximately 5.2 million cases, 470,000 hospitalizations, and 33,000 in-hospital deaths annually [7].

In pulmonary hypertension patients, infections are the leading cause of decompensation, and approximately 7% of the deaths in patients with PH are due to pneumonia [8]. There is also an increasing trend in PH mortality among the older population [9]. Patients with PH face a heightened risk of clinical deterioration triggered by respiratory infections due to their limited cardiopulmonary reserve [10]. Even mild viral infections can induce pulmonary vasoconstriction, exacerbate hypoxia, and precipitate acute decompensations that necessitate hospitalization [8].

Currently, the Centers for Disease Control and Prevention (CDC) recommends a single dose of RSV vaccine for all adults aged 75 years and older and adults aged 60–74 years who are at risk of severe RSV infection. Patients with chronic cardiovascular/lung disease, along with other chronic diseases, are at increased risk of severe RSV infection [11]. Despite these recommendations, patients with PH are not yet explicitly recognized as a high-risk subpopulation for RSV-related hospitalization.

It is unknown if the presence of PH in adults increases the risk of serious RSV infection compared to the rest of the adult population without PH. Given the significant morbidity and mortality associated with both RSV infection and PH, we aimed to assess if RSV infection increased hospitalization risk in individuals with PH compared to those without PH. The secondary aims of the study were the assessment of hospitalization-related complications, hospitalization risk due to various comorbidities, and PH-group-specific hospitalization risk.

## 2. Materials and Methods

### 2.1. Study Design

We performed a retrospective cohort study using the TriNetX database (https://trinetx.com, accessed on 7 April 2025). TriNetX is a global collaborative network that provides access to real-time de-identified patient information from electronic medical records, which includes diagnoses, procedures, laboratory values, and genomic information obtained from both inpatient and outpatient settings for approximately 122 million patients in approximately 100 healthcare organizations (HCOs) in the United States. It also includes prescription drug claims. Our study searched and analyzed data from 1 January 2015 to 7 April 2025.

**Figure 1:**
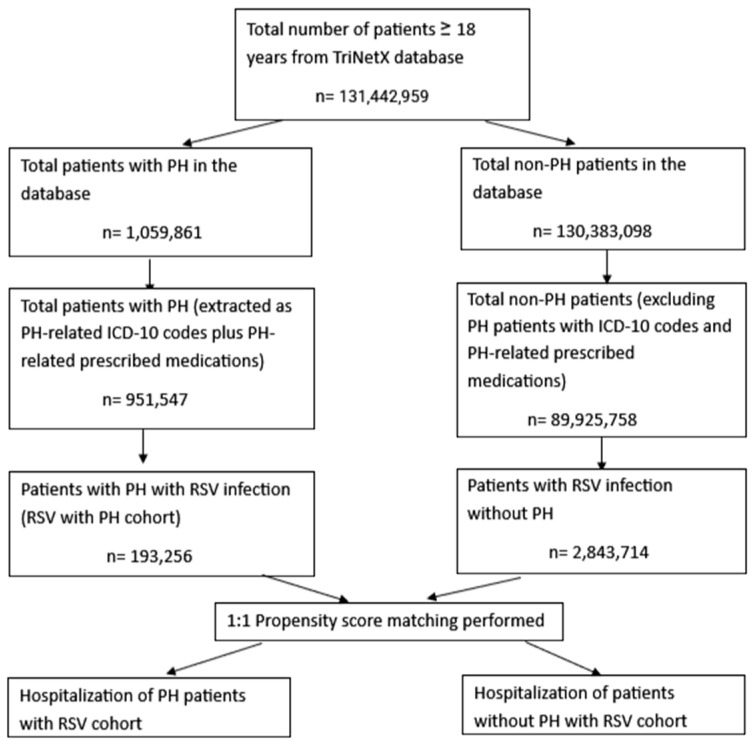
Flowchart of cohort selection in the RSV with PH and RSV without PH cohorts. Abbreviations: PH: pulmonary hypertension; RSV: respiratory syncytial virus; ICD-10: International Classification of Diseases, 10th edition.

Two cohorts were selected, namely the RSV with PH cohort and the RSV without PH cohort. Both these cohorts included patients >18 years of age. The RSV with PH cohort was selected based on International Classification of Disease, Tenth Revision, Clinical Modification (ICD-10-CM) codes for the various types of pulmonary hypertension plus RxNorm/VA:RE codes for ≥1 PH-related medication: ambrisentan, tadalafil, bosentan, macitentan, ambrisentan, riocoguat, sildenafil, treprostinil, epoprostenol, sotatercept, selexipag, furosemide, torsemide, carvedilol, metoprolol, sacubitril–valsartan, bronchodilators, anticholinergic bronchodilators, and sympathomimetic bronchodilators. The PH codes were selected from administrative claims data from multiple studies [12,13,14]. The RSV without PH group excluded all ICD-10-CM codes for PH along with PH-specific medications. Patients with RSV infection were identified using ICD-10-CM codes for RSV (J12.1, B97.4, J21.0, or J20.5) or Logical Observation Identifiers Names and Codes (LOINC) codes for a positive RSV laboratory test [15,16,17,18]. Supplementary Table S1 summarizes the codes used for this study. Since this database does not associate hospitalizations with a particular diagnosis, we included hospitalizations within one week of RSV diagnosis/positive test to determine RSV-related hospitalizations. This study included only RSV infections that occurred after a PH diagnosis. Figure 1 depicts a flowchart for the study’s patient selection.

The primary aim of the study was to assess the risk of hospitalization in the RSV with PH cohort compared with the RSV without PH cohort. Hospitalization was determined by the Current Procedural Terminology (CPT) code for hospital inpatient services or inpatient acute/non-acute, short stay, inpatient encounter within one week of RSV-positive lab result, or an ICD-10-CM code for RSV. The index date for both cohorts was chosen as the date of RSV infection in the database. We performed age-group-stratified subgroup analysis (18–49, >50, >60 years) to assess the hospitalization risk with PH in these age groups. Patients >60 years were chosen since the RSV vaccination is currently approved for patients >60 years of age with underlying chronic cardiac and lung diseases. We chose the 18–49 and >50 years age groups to assess if either of these groups might have a higher risk of hospitalization and therefore might benefit from earlier RSV vaccination.

Our study’s secondary aims were as follows:

A. Assess the risk of hospitalization-related complications, defined as the need for intensive care unit (ICU) care (identified as critical care services, CPT code 1013729), endotracheal intubation (CPT code 31500), vasopressor support into the peripheral vein/central vein (ICD-10 PCS codes 3E033XZ and 3E043XZ, respectively), or mortality (identified as deceased) within the next 30 days from the date of RSV-related hospitalization. We also assessed the risk of hospitalization for the composite outcome associated with these complications.
B. Assess the risk of hospitalization in RSV with PH groups 1–4 compared to the RSV without PH cohort. Supplementary Table S2 outlines the ICD-10-CM codes used for PH groups 1–4.
C. Assess the risk of hospitalization within the RSV with PH groups. The analysis was performed as individual groups compared with the other PH groups.
D. Assess the risk of hospitalization in the RSV with PH cohort with and without the following comorbid conditions: chronic lung disease (CLD), identified by the ICD-10-CM codes for asthma, chronic bronchitis, chronic obstructive lung disease, or bronchiectasis; cardiovascular disease (CVD), identified by the ICD-10-CM codes for ischemic heart disease, cerebrovascular accident, or peripheral vascular disease; diabetes mellitus (DM); and chronic kidney disease stage ≥3. The respective ICD-10-CM codes are listed in Supplementary Table S3.

### 2.2. Statistical Analysis

We conducted our analyses using TriNetX LLC which is a web-based cohort creation database and retrospective research tool. Our study included 91 HCOs. The baseline characteristics of the cohorts were presented as mean, standard deviation, and proportions. Propensity score matching was performed to account for potential confounders and ensure cohort comparability. Upon running the propensity score function, the patients were matched based on propensity scores to create a 1:1 ratio between cohorts using nearest neighbor (greedy) without replacement. A default caliper of 0.10 SD was used [19]. Demographic data, such as age at index event, gender, comorbid conditions like DM, CVD, and CKD, obesity, solid organ transplant status, alcohol-related disorder, and nicotine dependence, were selected and propensity matched as they have been associated with severe RSV infection [20,21,22,23]. *p*-values were calculated after propensity score matching, with <0.05 depicting statistical significance. The standard mean difference was also calculated, with <0.1 depicting good balance between groups. Outcomes were expressed as adjusted odds ratios (aORs) with 95% confidence intervals (CIs).

## 3. Results

### 3.1. Baseline Characteristics of the Study Sample

Of the 1,059,861 PH patients, 193,256 patients were noted to have RSV infection. There were 2,843,714 patients with RSV without PH. The patients with RSV with PH were predominantly white (64%), older in age (68.2 ± 15.3 vs. 49.7 ± 21.4), and 50.6% female compared to the RSV without PH cohort. The RSV with PH cohort had a significantly higher number of comorbidities, as listed in Table 1. We performed propensity score matching for the demographics and comorbidities between the two groups. All the variables were balanced well between the two cohorts, as noted by the *p*-value (<0.05) and/or mean standard difference (<0.1). Propensity score matching was also performed between the cohorts for connective tissue disorders (systemic lupus erythematosus, rheumatoid arthritis, systemic sclerosis, and overlap syndrome) because these disorders can lead to severe RSV infections and are also an etiological factor (approximately 15–25%) for group 1 PH [24,25,26,27].

### 3.2. Hospitalization Risk

There were 131,414 (68%) hospitalizations in the RSV with PH cohort compared to 1,059,066 (37.2%) in the RSV without PH cohort. The RSV with PH cohort displayed a higher odds of hospitalization risk compared to the RSV without PH cohort after propensity score matching (aOR 1.89, 95% CI 1.873–1.924, *p*-value 0.02). Interestingly, the age groups 18–49 years (aOR 3.08, 95% CI 2.939–3.229, *p*-value 0.03), >50 years (aOR 1.808, 95% CI 1.783–1.834, *p*-value 0.015), and >60 years (aOR 1.701, 95% CI 1.674–1.728, *p*-value <0.05) also demonstrated a higher risk of hospitalization after propensity score matching for the RSV with and without PH cohorts. Table 2 summarizes the above findings.

### 3.3. Hospitalization-Related Complications

Composite outcomes were noted in 31.3% and 21.6% of the RSV patients with and without PH, respectively. Mortality was noted in 8.2% of the RSV with PH cohort. A higher risk of composite outcomes (mortality, ICU care, need for intubation or vasopressor support) was noted in the RSV with PH group compared to the RSV without PH cohort after PSM (aOR 1.29, 95% CI 1.274–1.321, *p*-value <0.0001) within 30 days of hospitalization (Table 3). Similarly, the need for ICU care, intubation, and vasopressor support revealed higher risks (aOR 1.369, 1.375, and 1.553, respectively), which were statistically significant. Interestingly, there was less risk of mortality in the RSV with PH cohort, with an aOR of 0.920 (95% CI 0.892–0.948, *p*-value <0.0001).

### 3.4. Effect of Comorbid Conditions on Outcomes

The RSV with PH cohort was compared with and without certain comorbid conditions, namely, DM, CVD, CKD, and CLD. The data after propensity score matching demonstrated higher risks of hospitalization in the RSV with PH cohort with DM, CVD, CKD, or CLD compared to the RSV with PH cohort without DM, CVD, CKD, or CLD, which were all statistically significant (Table 4). The patients with CVD notably had a higher risk of hospitalization relative to the other comorbidities (aOR 5.1, 95% CI 4.78–5.43, *p*-value < 0.0001).

### 3.5. Hospitalization Risk Among PH Subgroups

We assessed the hospitalization risk among various PH WHO groups. Among groups 1, 2, 3, and 4, group 2 had a statistically significant higher risk of hospitalization compared to the other PH groups (aOR 1.771, 95% CI 1.697–1.848, *p*-value <0.0001). Notably, group 1 and group 3 had a lesser risk of hospitalization, which was statistically significant (Table 5). Group 4 did display a higher risk of hospitalization, but this was not statistically significant (aOR 1.02, 95% CI 0.781–1.328, *p*-value 0.892).

### 3.6. Comparison of Hospitalization Risk Among RSV with PH Subgroups vs. Non-PH Cohort

PH WHO groups 1, 2, 3, and 4 were compared with the RSV without PH group (Table 6). There were 16,505 (8.54%), 124,166 (64.25%), 49,628 (25.67%), and 1645 (0.85%) patients in the RSV with PH groups 1, 2, 3, and 4, respectively. After PSM, all groups noted higher risks of hospitalization, which were statistically significant.

## 4. Discussion

RSV is one of the most common causes of viral pneumonia. In some studies, patients admitted with influenza-like illness to the hospital noted an estimated prevalence of RSV of 12.5%, after influenza (18.7%) and rhinovirus/enterovirus (14.7%) [28,29]. PH has also been noted to have significant morbidity and mortality leading to hospitalization [30]. In our study, we assessed the risk of hospitalization in patients with PH who develop RSV infection and compared it to RSV-infected patients without PH. We found that among adult patients ≥18 years old, underlying PH increased their odds of hospital admission by 1.89 (95% CI 1.873–1.924, *p*-value 0.02). To our knowledge, this is the first study assessing the effect of RSV infection on adult patients with PH.

There are known risk factors for severe RSV infections, which include cardiac disease (congestive heart failure, coronary artery disease), lung disease (asthma, chronic obstructive pulmonary disease), cerebrovascular disease, chronic kidney disease, and diabetes mellitus [31,32]. Immunocompromised patients are at higher risk of acquiring RSV infection. Patients with hematopoietic stem cell transplants, lung transplants, and active chemotherapy demonstrate poorer outcomes [25,33]. Additional indicators of worse outcomes include smoking history, high altitude, leucopenia less than 100 cells/mm^3^, and history of total body radiation [33]. These conditions were therefore included in our study. We performed propensity score matching of the connective tissue disorders that are known to cause group 1 PH in approximately 15–25% of patients [26,27]. This reduced the selection bias between the two cohorts.

Interestingly, our data noted that patients in the age group 18–49 years had the highest risk of hospitalization, with an aOR of 3.08 (95% CI 2.939–3.229, *p*-value 0.03). This finding may be crucial, as currently, for patients between 18 and 59 years, the bivalent RSVpreF vaccine has been suggested but only if the patient is immunocompromised or has chronic lung, heart, or kidney disease, but not necessarily PH [34]. Our RSV with PH population was predominantly older than 50 years of age, similar to other studies [5,35].

Among patients admitted to the hospital with RSV infection, 10 to 30% have been found to require intensive care unit (ICU) admission, with up to 17% requiring mechanical ventilation [36,37]. The rate of ICU admission for RSV (24.3%) has been shown to be higher even than that of COVID-19 (17.3%) and influenza (16.8%) in a cohort of patients over the age of 60 years admitted with respiratory failure [37]. Our study demonstrated that after propensity score matching, in patients with RSV-related hospitalization, the presence of underlying PH (28.7% vs. 22.7%) was associated with increased odds of ICU admission by 1.369 (95% CI 1.343v1.395, *p*-value <0.0001), intubation/mechanical ventilation by 1.375 (95% CI 1.324–1.429, *p*-value <0.0001), and vasopressor support by 1.553 (95% CI 1.488–1.621, *p*-value <0.0001) within 30 days of hospitalization in comparison to the RSV without PH cohort. Accordingly, the composite outcome of hospital-related complications was increased, with an aOR of 1.369 (95% CI 1.343–1.395, *p*-value <0.0001). Although our study noted a mortality rate of around 7.5% in the RSV with PH cohort, similar to mortality rates in the RSV without PH cohort of around 6–8% (also noted in the study by Colosia et al.), it did not reflect increased mortality in the former cohort (aOR 0.920, 95% CI 0.892–0.948, *p*-value <0.0001) [36]. This could be due to a smaller follow-up period of 30 days from hospitalization, a longer length of stay, or earlier intervention for management in this cohort due to much more severe illness.

Cardiovascular disease has been linked to increased RSV incidence and complications. Children with congenital heart disease and cardiomyopathy have been found to have increased hospital admissions and severe RSV infections [38]. Loubet et al. found that around 45% of patients with lab-confirmed RSV had chronic heart disease [25]. Duncan et al. demonstrated that among patients admitted to the inpatient setting with RSV infection, 28% had congestive heart failure (CHF) as compared to 0% in the outpatient setting, with a trend toward more coronary artery disease (CAD) (25% vs. 8%) [39]. Similar rates of CHF (21%) and coronary artery disease (28%) were observed by Dowell et al. in 57 patients admitted with RSV pneumonia [40]. McCraken and colleagues also determined that patients with underlying heart disease were more likely to develop a severe hospital outcome (OR 4.1; 95% CI 1.9–8.8) [41]. Even among patients with COPD, CHF was the only significant risk factor for developing severe RSV infection [42]. However, PH was not assessed as a potential risk factor in any of these studies. Some studies have noted that almost 50% of patients with heart failure with preserved ejection fraction have PH [43,44]. Our study demonstrated that among patients with RSV with PH, concomitant CVD had a significantly higher risk of hospitalization (aOR 5.102, 95% CI 4.787–5.438, *p*-value <0.0001) compared to RSV with PH patients without CVD. Of note, we did not include heart failure in our analyses for CVD, as heart failure denotes PH disease progression [43]. The presence of chronic lung disease, chronic kidney disease, and diabetes mellitus increased the odds of hospitalization in PH patients with RSV infection. These comorbidities have previously been elucidated as risk factors for severe RSV infection [5,32].

Pulmonary hypertension in itself is an evolving paradigm, with recent changes to the diagnostic criteria and newer medications being approved for therapy [45,46]. Most patients are categorized as group 2 (69%) as compared to group 1 (15%), group 3 (9%), and groups 4 and 5 (3% each) [47]. In our study, similarly, patients with group 2 PH had a higher risk of hospitalization when compared to other PH WHO groups (aOR 1.771, 95% CI 1.697–1.848, *p*-value <0.0001), further emphasizing the importance of RSV prevention in patients with underlying heart disease. Interestingly, when we compared each PH group with RSV individually with the RSV without PH cohort after propensity score matching, all groups revealed a statistically significant increase in hospitalization risk. Though the sample size of these groups was small compared to the RSV without PH cohort, these findings depict a need for further studies to assess if PH by itself acts as an independent risk factor affecting RSV severity. Some studies have noted elevated serum cytokines (tumor necrosis factor-alpha, interferon-gamma, and interleukin-1beta, -2, -4, -5, -6, -8, -10, -12p70, and -13) in group 1 PH patients [48,49]. This cytokine dysregulation may be responsible for exacerbating RSV-induced inflammation as a possible mechanism. Reciprocal RSV-induced PH has previously been demonstrated in mouse models, which could also help decipher the pathogenesis of PH in RSV [50].

Currently, three different vaccines are approved by the US Food and Drug Administration (FDA). The mRNA RSV vaccine (mResvia) is approved for people older than 60 years, while an adjuvanted monovalent RSV vaccine (Arexvy) is approved for people between 50 and 59 years, and for people 18–59 years of age, a bivalent PreF vaccine (Abrysvo) is approved [51]. On June 26, 2024, the Advisory Committee on Immunization Practices (ACIP) recommended one of these RSV vaccines for all adults aged ≥75 years, for adults aged 60–74 years with the above risk factors, and for those with moderate to severe immunocompromise [52]. These recommendations were made in agreement with the Centers for Disease Control and Prevention (CDC), which, in addition to the above-mentioned risk factors, includes end-stage renal disease, morbid obesity, chronic liver disease, chronic hematologic conditions, neurological or neuromuscular conditions, nursing home residence, and medical judgment. Thus, the CDC recommends a one-time RSV vaccine in a larger group of individuals, either inpatient or outpatient [53].

Despite this, few studies have noted that for the year 2024–2025, in the 51.8% of facilities that voluntarily elected to report RSV vaccination, only 17.9% of patients received it, highlighting the large population at risk but without access to the RSV vaccine [54]. In multiple studies of vaccine efficacy in patients over 60 years of age, hospitalization rates have been reduced by 70–80% [55,56]. Although the RSV vaccines are well tolerated, safety data in patients >65 years revealed an increased risk of developing Guillain–Barré syndrome (incidence rate ratio 2.02; 95% CI 0.93–4.40) within the first 42 days of receiving the vaccine [57]. Despite the potential increase, the risk of Guillain–Barré syndrome following RSV vaccination in older adults remains rare, with the benefit outweighing the risk [58]. Patients under 60 years have not demonstrated this risk.

Our study has some limitations. This is a TriNetX-database-based retrospective cohort study in which data was not available from physician notes for WHO functional class for severity assessment, as well as for the right heart catheterization procedure for hemodynamics (considered the gold standard for PH diagnosis). Echocardiogram data was also sparse. We therefore used ICD-10 codes for pulmonary hypertension in combination with PH-specific medications to improve the positive predictive value and specificity of our study [12,13,14]. We used propensity score matching to reduce confounding, but there might be some residual confounding due to the inherent nature of electronic health records data. Only patients with an outpatient/inpatient RSV-positive test and/or an ICD-10-CM code were included in our study to improve sensitivity and prevent misclassification bias. We also did not assess the impact of other co-existing viral infections in our study population. Finally, due to the real-time availability of extensive and diverse data, we were able to perform our primary and secondary data analysis on a large sample size.

## 5. Conclusions

Our retrospective cohort study demonstrated that PH is a potential risk factor for severe RSV infections. The administration of newer RSV vaccines in PH patients of all age groups, >18 years of age, therefore might prove beneficial. Further research needs to be performed to understand the underlying mechanism to reduce the risk of RSV-related outcomes in this susceptible population.

## Figures and Tables

**Table 1:** Patient demographics and comorbidities for the PH–RSV and RSV only cohorts before and after propensity score matching.

	Before Propensity Score Matching			After Propensity Score Matching			Std.dif
	RSV with PH (*n* = 193,256)	RSV Without PH (*n* = 2,843,714)	*p*-Value	RSV with PH (*n* = 188,903)	RSV Without PH (*n* = 188,903)	*p*-Value	
**Demographics**							
**Age at Index, Mean ± SD**	**68.2 ± 15.3**	**49.7 ± 21.4**	**<0.001**	**68.2 ±15.3**	**69.5 ± 14.6**	<0.001	0.088
Female, *n* (%)	96,537 (50.6%)	1,454,243 (56.4%)	<0.001	95,478 (50.5%)	94,921 (50.2%)	0.07	0.006
White	122,159 (64.0%)	1,649,239 (63.9%)	0.527	121,265 (64.2%)	124,174 (65.7%)	<0.001	0.032
Black or African American	34,140 (17.9%)	404,163 (15.7%)	<0.001	33,436 (17.7%)	31,666 (16.8%)	<0.001	0.025
Hispanic or Latino	13,814 (7.2%)	320,991 (12.4%)	<0.001	13,693 (7.2%)	12,741 (6.7%)	<0.001	0.02
**Comorbidities, *n* (%)**							
Diabetes mellitus	85,058 (44.6%)	426,208 (16.5%)	<0.001	83,604 (44.3%)	84,615 (44.8%)	0.001	0.011
Ischemic heart disease	99,044 (51.9%)	328,169 (12.7%)	<0.001	97,278 (51.5%)	97,367 (51.5%)	0.772	0.001
Chronic lower respiratory diseases	98,729 (51.7%)	596,064 (23.1%)	<0.001	96,995 (51.3%)	97,442 (51.6%)	0.146	0.005
Cerebral infarction	21,530 (11.3%)	93,726 (3.6%)	<0.001	21,131 (11.2%)	21,282 (11.3%)	0.436	0.003
Chronic kidney disease (CKD)	76,033 (39.8%)	228,903 (8.9%)	<0.001	74,347 (39.4%)	72,223 (38.2%)	<0.001	0.023
Alcohol-related disorders	16,765 (8.8%)	136,459 (5.3%)	<0.001	16,501 (8.7%)	16,460 (8.7%)	0.813	0.001
Nicotine dependence	47,420 (24.8%)	407,835 (15.8%)	<0.001	46,765 (24.8%)	47,901 (25.4%)	<0.001	0.014
Obesity, unspecified	62,099 (32.5%)	364,533 (14.1%)	<0.001	60,750 (32.2%)	58,805 (31.1%)	<0.001	0.022
Liver transplant status	2074 (1.1%)	6276 (0.2%)	<0.001	1870 (1%)	1783 (0.9%)	0.148	0.005
Kidney transplant status	4930 (2.6%)	15,060 (0.6%)	<0.001	4591 (2.4%)	4331 (2.3%)	0.005	0.009
Stem cell transplant	1144 (0.6%)	11,343 (0.4%)	<0.001	1126 (0.6%)	1225 (0.6%)	0.041	0.007
Human immunodeficiency virus (HIV) disease	1556 (0.8%)	1460 (0.8%)	0.079	1556 (0.8%)	1460 (0.8%)	0.079	0.006
**Connective Tissue Disorders**							
Systemic lupus erythematosus (SLE)	3609 (1.9%)	3289 (1.7%)	0.963	3609 (1.9%)	3289 (1.7%)	<0.001	0.013
Rheumatoid arthritis	8924 (4.7%)	8930 (4.7%)	<0.001	8924 (4.7%)	8930 (4.7%)	0.963	<0.001
Systemic sclerosis (scleroderma)	1756 (0.9%)	1319 (0.7%)	<0.001	1756 (0.9%)	1319 (0.7%)	<0.001	0.018
Other overlap syndromes	848 (0.4%)	630 (0.3%)	<0.001	848 (0.4%)	630 (0.3%)	<0.001	0.018

Abbreviations: PH: pulmonary hypertension; RSV: respiratory syncytial virus; Std.diff: mean standard difference.

**Table 2:** Risk of hospitalization in the RSV with PH cohort and the RSV without PH cohort after propensity score matching, expressed as aOR with 95% CI.

Age Groups	RSV with PH Group	RSV without PH Group	aOR	95% CI	*p*-Value
≥18 years	128,458 (68%)	99,777 (53%)	1.89	1.873, 1.924	0.02
18–49 years	9472 (63%)	5363 (35.8%)	3.080	2.939, 3.229	0.03
>50 years	115,321 (68.4%)	91,871 (54.5%)	1.808	1.783, 1.834	0.015
>60 years	88,890 (67.3%)	72,332 (54.8%)	1.701	1.674, 1.728	<0.05

Abbreviation: aOR: adjusted odds ratio.

**Table 3:** Risk of hospitalization-related complications between the RSV with PH cohort and the RSV without PH cohort after propensity score matching.

Hospital Complications	RSV with PH Group	RSV without PH Group	aOR	95% CI	*p*-Value
Composite	36,041 (32%)	29,987 (27%)	1.298	1.274, 1.321	<0.0001
Mortality	8434 (7.5%)	9111 (8.1%)	0.920	0.892, 0.948	<0.0001
Critical care services	32,193 (28.7%)	25,494 (22.7%)	1.369	1.343, 1.395	<0.0001
Intubation	6580 (5.9%)	4862 (4.3%)	1.375	1.324, 1.429	<0.0001
Vasopressor support	5500 (4.9%)	3604 (3.2%)	1.553	1.488, 1.621	<0.0001

**Table 4:** Risk of hospitalization in the RSV with PH cohort in patients with and without comorbid conditions after propensity score matching, expressed as aOR with 95% CI.

RSV with PH Group with Comorbidity	Number of Patients	aOR	95% CI	*p*-Value
With DM	16,760 (76.6%)	1.083	1.078–1.089	<0.0001
Without DM	15,627 (71.4%)			
With CVD	26,367 (74.3%)	5.102	4.787–5.438	<0.0001
Without CVD	23,708 (66.8%)			
With CLD	22,860 (75.8%)	2.628	2.466–2.801	<0.0001
Without CLD	22,095 (73.2%)			
With CKD	51,749 (76.3%)	3.076	2.894–3.269	<0.0001
Without CKD	48,885 (72.1%)			

Abbreviations: aOR: adjusted odds ratio; CVD: includes ischemic heart disease, mitral valve stenosis, congenital heart disease, cerebral infarction, peripheral vascular disease, and arterial thrombosis; DM: diabetes mellitus; CLD: chronic lung disease, including chronic bronchitis, COPD, asthma, and bronchiectasis; CKD: chronic kidney disease, including CKD stage >3 or ESRD.

**Table 5:** In-group RSV with PH comparisons and risk of hospitalization after propensity score matching, expressed as aOR with 95% CI.

In-Group RSV with PH Comparisons	aOR	Confidence Interval	*p*-Value
Group 1 vs. other PH groups	0.735	0.686–0.787	<0.001
Group 2 vs. other PH groups	1.771	1.697–1.848	<0.0001
Group 3 vs. other PH groups	0.897	0.874–0.922	<0.001
Group 4 vs. other PH groups	1.02	0.781–1.328	0.892

Abbreviations: aOR: adjusted odds ratio; PH: pulmonary hypertension.

**Table 6:** Hospitalization risk in the PH WHO subgroups vs. the RSV without PH cohort after propensity score matching, expressed as aOR with 95% CI.

RSV with PH Subgroups (Number of Patients)	RSV without PH (*n* = 2,843,714)		
	aOR	95% CI	*p*-Value
Group 1 (16,505)	2.576	2.437- 2.722	< 0.001
Group 2 (124,166)	3.892	3.804- 3.982	<0.0001
Group 3 (49,628)	3.741	3.644–3.841	<0.001
Group 4 (1645)	3.872	3.181–4.713	<0.0001

Abbreviations: aOR: adjusted odds ratio.

## Data Availability

Data is contained within the article.

## Supplementary Materials

The following supporting information can be downloaded at: https://www.mdpi.com/article/10.3390/biomedicines13092272/s1, Table S1: List of ICD-10-CM, RxNorm, and LOINC codes used in cohort identification and outcome definition; Table S2: List of WHO PH groups with their respective ICD-10-CM/RxNorm codes; Table S3: List of ICD-10-CM codes used for identification of various comorbid conditions.
