# Supplementary material for: The Impact of Pulmonary Hypertension on Hospitalization Risk in Adults with Respiratory Syncytial Virus Infection
Source: Biomedicines. 2025 Sep 16;13(9):2272. doi: 10.3390/biomedicines13092272 (PMC12467006; doi:10.3390/biomedicines13092272)
Supplement: Supplementary file 1 [file biomedicines-13-02272-s001.zip › biomedicines-3753080-supplementary.pdf]

biomedicines

MDPI

Supplementary information

Table S1. List of ICD-10-CM, RxNorm, and LOINC codes used in cohort identification and outcome definition.

Pulmonary Hypertension Codes	ICD-10-CM Code
Primary pulmonary hypertension	I27.0
Secondary pulmonary arterial hypertension	I27.21
Other secondary pulmonary hypertension	I27.29
Pulmonary hypertension due to left heart disease	I27.22
Pulmonary hypertension due to lung disease and hypoxia	I27.23
Other pulmonary heart disease	I27
Pulmonary heart disease, unspecified	I27.9
Chronic thromboembolic pulmonary hypertension	I27.24
Pulmonary hypertension, unspecified	I27.20
**Medication**	**RxNorm code/VA code**
Ambrisentan	358274
Tadalafil	358263
Bosentan	75207
Macitentan	1442132
Ambrisentan	358274
Riocoguat	1439816
Sildenafil	136411
Treprostinil	343048
Epoprostenol	8814
Sotatercept	2678930
Selexipag	1729002
Furosemide	4603
Torsemide	38413
Carvedilol	20352
Metoprolol	6918
Sacubitral	1656328
Valsartan	69749
Bronchodilators	VA:RE100
Anticholinergic bronchodilators	VA:RE105
Sympathomimetic bronchodilators	VA:RE102
**RSV Infection**	**ICD-10-CM code/LOINC code**
Respiratory syncytial virus pneumonia	J12.1
Respiratory syncytial virus as the cause of diseases classified elsewhere	B97.4
Acute bronchitis due to respiratory syncytial virus	J20.5
Acute bronchiolitis due to respiratory syncytial virus	J21.0
Respiratory syncytial virus positive test	31950-9; 33045-6; 40988-8; 76089-2; 82176-9; 92131-2; 5876-8; 60271-4; 72885-7; 5877-6; 68966-1; 30075-6; 30076-4; 85479-4; 77023-0; 77022-2; 92957-0; 80597-8

Abbreviations: ICD-10-CM: International Classification of Diseases, Tenth Revision, Clinical Modification; CPT – Current Procedural Terminology.

 https://doi.org/10.3390/biomedicines13092272

Table S2. List of WHO PH groups with their respective ICD-10-CM/RxNorm codes.

PH WHO Groups	ICD-10-CM Code or RxNorm Code
***Group 1 PH**	
Primary pulmonary hypertension	I27.0
Secondary pulmonary artery hypertension	I27.21
Pulmonary artery hypertension, unspecified	I27.20
plus	
Group-1-specific PH medications (from Supplementary Table 1)	
***Group 2 PH**	
Pulmonary hypertension due to left heart disease	I27.22
Pulmonary hypertension, unspecified	I27.20
plus	
Cardiac medications from Supplementary Table 1	
plus	
Heart failure	I50
Rheumatic mitral valve disease/mitral stenosis	105/105.5/105.2
Non-rheumatic mitral valve stenosis	I34/134.2
Non-rheumatic aortic valve disorders	I35
***Group 3 PH**	
Pulmonary hypertension, unspecified	I27.20
Pulmonary hypertension due to lung disease and hypoxia	I27.23
plus	
Pulmonary medications from supplementary table 1	
plus	
Other chronic obstructive pulmonary disease, morbid obesity, pulmonary fibrosis, obstructive sleep apnea	J44/E66.2/J84.10/G47.3
***Group 4 PH**	
Chronic thromboembolic pulmonary hypertension	I27.24
plus	
Medications for thromboembolism from Supplementary Table 1 along with warfarin	1439816/11289

Abbreviations: ICD-10-CM: International Classification of Diseases, Tenth Revision, Clinical: *Other PH groups were added to the exclusion criteria for the respective PH group selection.

Table S3. List of ICD-10-CM codes used for identification of various comorbid conditions.

Co-morbid Conditions	ICD-10-CM Code
**Diabetes mellitus**	E08-E13
**Chronic lower respiratory diseases**	J40-J47
**Cardiovascular diseases**	
Ischemic heart disease	I20-25
Other peripheral vascular diseases	I73

Arterial embolism and thrombosis	I74
Cerebral infarction	I63
**Chronic kidney disease stage 3 or higher**	
Chronic kidney disease, stage 3	N18.3
Chronic kidney disease, stage 4	N18.4
Chronic kidney disease, stage 5	N18.5
End-stage renal disease	N18.6

Abbreviations: ICD-10-CM: International Classification of Diseases, Tenth Revision, Clinical Modification.
